# Medical and Philosophical Intelligence

**Published:** 1816-04

**Authors:** 


### MEDICAL AND PHILOSOPHICAL INTELLIGENCE.

WE copy the following from Dr. Thompson's Annals of Philosophy, not venturing our remarks till we see the paper at length. All our attention, during the reading of the paper at Somerset Place, was insufficient to enable us to combine the reasoning with the experiments in a manner satisfactory to ourselves:—

*Royal Society.*—At a meeting of this society, on Thursday the 25th of January, a paper by Dr. WILSON PHILIPS was partly read, containing experiments on the nervous influence in secretion. In two former papers he had shown that the circulation of the blood and the action of the muscles were independent of the nervous influence, and that this influence only acted on the muscles like any other stimulus. But the case is very different with the secretions. Whenever the nervous influence is interrupted the secretion is at an end. Several rabbits had the eighth pair of nerves divided, and in all of them the parsley, which they ate after the operations, remained in the stomachs quite unaltered, and exactly resembled parsley chopped small with a knife. The stomach was always much distended, and a portion of the food was contained in the œsophagus. This was owing to the unsuccessful attempts which the animal made to vomit, which always follow the division of the eighth pair. The animal soon shows a violent dyspnœa, and seems to die at last of suffocation.

Since the experiments of Galvani on animals, it has been a favourite opinion of many physiologists, that the nervous influence is the same with galvanism. To put this to the test of experiment, a portion of the hair of a rabbit opposite to the stomach was shaved, a shilling tied on it, the eighth pair was divided, and the extremities of the nerve coated with tinfoil. These were connected with a galvanic battery of forty-seven pairs of plates, four inches square. The trough was filled with a liquid composed of one part muriatic acid and seven parts water. This action was kept up for twenty-six hours. No dyspnœa took place; and after death the food in the stomach was found as much digested as in the stomach of a healthy rabbit which had eaten food at the same time. The smell of the parsley was destroyed, and the smell existed which is peculiar to the stomach of a rabbit during digestion. This experiment was several times repeated with the same result. So that it appears that the galvanic energy is capable of supplying the place of the nervous influence, so that while under it the stomach digests food as usual.

Dr. Philips likewise made a number of experiments to show that heat is a secretion from the blood, produced by means of the nervous energy. When new drawn blood is subjected to the action of the galvanic battery, it continues several degrees hotter than blood not subjected to the same process.

On Thursday, the 1st of February, Dr. Wilson Philips' paper was continued: he considers it, as proved by his experiments, that the ganglia communicate to the nerves proceeding from them the general influence of the brain and spinal marrow. Nerves proceeding from them supply all the involuntary muscles. But if this be the case, it will be asked, how comes the digestive power of the stomach to be destroyed by cutting the eighth pair of nerves, seeing that the stomach is supplied with nerves from ganglia? The eighth pair coming from the largest portion of the nervous matter, possesses the greatest influence; but the digestive power of the stomach is weakened likewise by the interruption of the nerves proceeding from ganglia. This he proved by destroying part of the lower portion of the spinal marrow of different rabbits. In every case the digestive power of the stomach was impaired or destroyed; the urinary bladder and rectum lost the power of discharging their contents; and paralysis of the lower extremities ensued, and a great degree of cold took place. The heat of one rabbit before death sunk as low as 75°. Though the power of the stomach, as an organ of digestion, is destroyed by cutting the eighth pair of nerves, still its muscular power remains; but it does not act as usual, because the stimulus of digested food is wanting; or it acts so as to throw the food out of the stomach the wrong way, in consequence of the unnatural stimulus of undigested food.

On Thursday, the 8th of February, Dr. Wilson Philips' paper was concluded. He shewed that the heat of animals was in all probability owing to the nervous energy. He finished his paper with a general view of the facts which he had established in the three papers which he had laid before the Royal Society. The muscular energy depends upon the particular structure of the muscles; the nervous system is supported by the sanguiferous; but the sanguiferous can act without the influence of the nervous system. Secretion and animal heat are entirely dependent upon the nervous system.

system. Hence the muscles cannot for any length of time continue to exert their energy if the nervous influence be cut off. The nervous influence appears the same with the galvanic energy.

Mr. Todd, a surgeon in the Royal Navy, presented an account of his observations made on the Torpedo at the Cape of Good Hope. The peculiar organs of this animal have been described by the late Mr. Hunter. Mr. T. found that when the electric organs are often excited they lose their power, and the animal dies much sooner. Its first strokes are always the most violent, and grow gradually more and more feeble until quite exhausted, and then the animal dies. The author cut open the little tubes or electric organs in the breast; and by this process the animal lost its electric powers, but continued to live longer than those whose electricity was entirely exhausted. The torpedos subjected to these experiments were smaller than those found in the northern seas, being only from five to eight inches long, and from three to five broad. They were caught by the sailors, when fishing in the usual manner, while the Lion lay at anchor off the Cape. Some of the torpedos manifested a kind of reluctance to give shocks; others parted with them very freely: hence the author is inclined to believe that it requires a considerable effort in the animal to give shocks, and that it shortens its life. The torpedos were kept in casks of salt-water, in which they lived from two to five days.

On the 21st of February, a short paper by Sir Everard Home was read, containing some observations on the structure of the feet of some lacertæ, particularly the *gecko*. Sir Joseph Banks, who suffers nothing to escape his observation, noticed, while in Batavia, that the gecko is a very familiar inmate of the houses; and that it could run along their smooth ceilings, having its back downwards, with the greatest ease, contrary to the laws of gravity. He mentioned this circumstance to Sir Everard, and also supplied him with a large one weighing three ounces, in order that he might examine the structure of its feet. The result of his inquiry is, that the feet of the gecko have some resemblance to the *actinia* of those fish which adhere to the sides of ships; that they, at every step, form a partial vacuum below them, which thus enables them to run with their back downwards.

On this evening their Imperial Highnesses the Archdukes John and Lewis, of Austria, brothers of the Emperor of Austria, having been elected at a previous meeting, were regularly introduced as fellows of the Royal Society of London for improving natural knowledge.—*Philos. Mag.*

---

*Medical Society of London, Bolt Court.*—The anniversary of this Society was celebrated on the 8th of March, when the oration was spoken by Dr. Clutterbuck. The orator began by teaching his hearers to expect a general view of the medical science, rather than an examination of any select article. In this they were not disappointed. After a very elegant and classical history of medicine from from its earliest date, including the great father of physic, with Celsus, Galen, and also the writers of the middle ages; the improvements under Sydenham, Stahl, Boerhaave, and others, were pointed out; and an impartial history given of the Cullenian school. The orator then adverted, with a due sense of respect, not however unmixed with the gravity of true irony, to the practitioners of the present day and their immediate predecessors. The merits of the two Hunters were fairly appreciated; but the division which John has introduced of the various morbid poisons formerly considered as different forms of the venereal disease, were hinted at with more scepticism than we expected. The subject readily introduced the too indiscriminate exhibition of mercury in all disorders, and the minute stercoraceous examinations which seem to render the physician if not ridiculous, at least as empirical as those who undertake to cure all diseases by examining the urine.

The oration was throughout interesting, perspicuous, and eloquent. It was received as might be expected by a medical audience, and an unanimous resolution passed requesting it to be printed.

The following gentlemen were returned officers and members of the council for the year ensuing, viz.—

President—J. Adams, M.D. F.L.S.

Vice-Presidents—W. Babington, M.D. F.R.S.; R. Temple, M.D.; W. Norris, Esq.; R. Saumarez, Esq.

Treasurer—J. Andree, Esq.

Librarian—J. Hancock, M.D.

Secretary for Foreign Correspondence—C. Taylor, M.D.

Registrar—T. J. Pettigrew, F.L.S.

Council—Drs. Walshman, Clutterbuck, Hamilton; Messrs. E. Leese, B. Atkinson, A. Mathias, J. Taunton, J. Sauer, T. J. Pettigrew, G. Damant, M. Bartlett, J. Harding, E. Sutliffe, W. Edenborough, J. Stevenson, W. K. Griffith, E. Austin, W. Edwards, J. H. Hooper, J. Dunlap, J. Jones, J. Seaton, S. Sawrey, P. Heaums, T. Wheelwright, K. Johnston, S. Griffith, T. Whateley, J. Beveridge, B. Brown.

---

The Royal Institute of France have published several ingenious chemical papers. We shall take a future opportunity of compressing the result of the whole in a manner more interesting, because less tedious, to our medical readers.

---

We have received from Newcastle, a Memorial on the Apothecaries' Bill, signed by most of the professional gentlemen of that part. The paper is useful, inasmuch as it contains whatever may be found objectionable in the bill. Most of these objections have indeed already appeared in a more scattered form, but this does not supersede the usefulness of the present work, which we earnestly recommends to the perusal of the faculty at a time when the College of Surgeons are negociating a parliamentary charter. We are, however, obliged to add, that this memorial is tediously

1 prolix; prolix; and, in some parts, appears to us not perfectly consistent with the dignity we should expect from so many truly respectable signatures. The title is also quaint, as a memorial is usually presented to some persons. This seems addressed to no one, not even to the public at large, excepting by presumption.

---

*Observations on the Generation of the Guinea Worm;* by George Thomas Heath, late Surgeon of his Majesty's Ship Psyche.—As it appears by Dr. Chisholm's paper on Guinea-worms, published in your last number, that the *mode* in which those animalculæ obtain admission in the human frame, is by no means clearly ascertained, I beg leave to submit the following facts, which may, perhaps, somewhat elucidate this obscure subject.

His Majesty's ship Psyche had been in Bombay dock and harbour during the first three months of the year 1808. In April she sailed from thence, and in three months afterwards, being then in the Persian gulf, two of the men were found affected by guinea-worms; others also complained at various intervals; and, in the course of a twelvemonth from quitting Bombay, eight men in the whole had them.

From this period (April 1809) to the end of the year, seventy-four persons were put on the sick-list with those worms, some of whom had them two, three, four, and even five times, the worms issuing, at different periods, from various parts of the trunk and limbs. The length of the worms varied from nine to forty-two inches. Not one of the officers was at any time affected by them.

The alarming prevalence of those worms, the very distressing effects and long confinement in many instances induced, excited considerable interest on board, and caused every inquiry to be made, in order to obtain information on a subject of such importance.

As officers and men had been equally on shore while the ship was in dock at Bombay, had drank of the *same water* there, and of the same stock taken to sea, we could not believe that the worms, or their embryos, were introduced through the medium of the water drunk. The only way in which we could account for their introduction and exclusive confinement to the seamen, was by supposing that the worms, in a minute or embryo state, penetrated the exposed skin of the men, who usually went about without any other coverings than a hat, a shirt, and trowsers; while the officers, who were properly clad, and generally wore boots or gaiters, were thus shielded against them.

The idea of contagion having been suggested, it may not be improper to add, that three drafts of recently impressed men, and a detachment of his Majesty's 56th regiment, were received on board while the worms were most prevalent, without any one of them receiving the disease; and I may add, that two of the Psyche's men were discharged in two other of his Majesty's ships, where they both had guinea-worms, without communicating the disease to any other person.—*Edin. Journ.*

On

*On Safe-Lamps for Mines.*—In the month of November last I had the pleasure to communicate to you the result of several successful experiments, made in the presence of the Literary and Philosophical Society here, with the safe-lamp invented by Mr. Stephenson, which, I am happy to add, has been since used in the most dangerous parts of some coal-mines without any accident having occurred. On Tuesday the 6th instant, Sir H. Davy's recently improved lamp, the flame of which is encompassed by wire-gauze, was also exhibited by a professional gentlemen who had previously tried it at Walls End and Hebburn collieries; and its merits appear to be still greater than those of Mr. Stephenson's. The lamp being suspended in a vessel of glass, open at the top, and the carburetted hydrogen admitted from below, the bright flame of the wick nearly disappeared, but the cylinder of wire-gauze was filled with a feeble but steady greenish light. On a greater volume of inflammable air being thrown in, the flame gradually died out. Results more satisfactory could not be expected nor wished for, particularly when we were assured that these accorded with numerous trials made in the most hazardous drifts of our coal-mines.

Notwithstanding all that has been lately said in some of the periodical publications, respecting the obstinacy of the viewers employed here, and the stupidity of their under-agents and pitmen, you may depend upon it that these safe-lamps are hailed by this class of people as a most fortunate discovery, which will soon be adopted by them in every mine infested with fire-damp. And, could a mode be struck out of preventing inflammation taking place by means of a furnace placed at the bottom of the upcast shaft, to accelerate the circulation of air through the workings, little would be wanting to render the occupation of the collier as safe at least as that of the persons employed in lead and copper mines.—

Your most obedient servant, N.

*Newcastle-upon-Tyne, Feb.* 16, 1816.

[*From the Philosophical Magazine and Journal.*]

---

*On a Method of making Ship Lanterns with Mica and Wire;* by M. Rochon.*—The great fragility of glass does not allow of its being employed in every sort of lantern, lamp, &c. In the navy it is necessary that the watch-lights, those of the powder-room, &c. should transmit the light through horn, or other substance capable of resisting great shocks. This material is at present sufficiently plentiful, and is very well manufactured, in France; but, as it fell short in the magazines at the beginning of the revolution, M. Rochon supplied its place for the ship-lanterns by a net-work of wire, of a large mesh, covered with a light coat of transparent isinglass. This artificial horn was at that time of great service in the navy.

* From the Annales des Arts et Manufactures.

The

The arrival of an American vessel, having on board several pieces of foliated mica, perfectly transparent, suggested to M. Rochon the idea of employing it in the place of glass or horn, in preference to isinglass and copal varnish. This mineral is found in abundance in the quarries of granite in the environs of Newport, in North America.—*Philos. Mag.*

---

*On Nitrate of Silver as a Test of Arsenic.*—An ingenious student at Guy's Hospital suggested to Dr. Marcet, that, when nitrate of silver is mixed with a solution containing an alkaline phosphate, a yellow precipitate is thrown down, similar in appearance to arseniate of silver. Hence an ambiguity in the mode of detecting arsenic in the liquid contained in the stomach, where an alkaline phosphate may very well be present. This fact induced Dr. Marcet to examine the subject anew. The shade of the two salts is not quite the same. Yet in juridical cases other tests may be requisite to be assured of the presence of arsenic. The addition of sulphate of copper and potash, and the formation of Scheele's green, affords a very satisfactory confirmation. But the best mode of proceeding is to mix the supposed arsenic of silver with a little potash and charcoal powder, and expose it to heat in a glass tube. A pellicle of metallic arsenic will be obtained on the inside of the tube, unless the quantity of arsenic present be very minute indeed.

---

*St. Helena.*—The late Dr. Roxbourgh, while at St. Helena, where he spent several months, drew up a flora of that island. He found in it fifty-six species, fifty of which were peculiar to the island, having been observed no where else. Not a single new genus occurred.

---

*Rumford Prize.*—The council of the Royal Society has voted the Rumford prize to Dr. Wells, for his Essay on Dew.

---

*Caterpillars in Switzerland.*—A very singular phenomenon has lately taken place in Switzerland, at the distance of about nine miles from Lauzanne. The whole surface of the snow is covered with a species of caterpillar, different from any which are usually observed in that country. These animals appear dead; but, when brought near the fire, they soon recover animation.—*Annals of Philosophy.*

---

FOREIGN INTELLIGENCE.

Dr. Vogel, in Glogau, has cured the epilepsy, without any relapse, by the application of the Succus expressus Galii Moluginis.

The same physician has cured insanity by the following remedy: Aquæ Melissæ ℥iv. Extracti Angelicæ, Extracti Dulcamaræ āā ʒj. Extracti Belladonæ gr. iv. Spiritus Nitri dulcis ʒij.

Remaining weakness of memory, and of the power of judgment, were then removed by ℞. Spiriti Rutae ℥ij. Balsami Peruvian ʒij. to be rubbed in on the spine early every morning.

Dr. Krosck, in Greiffenberg, recommends the Liquor Stypticus Loffii, from eight to twelve drops, diluted in six ounces of water, to be given a table-spoonful every hour after an abortion, or in lochiæ after delivery. The preparation of this liquor is difficult; but its efficacy is quite equal to that difficulty.

In great sensibility of the feet, sand-baths prepared for them were found by Dr. Purlitz of great service.

Dr. Busser, in Wohlau, cured an insanity, arising on the seventh day after delivery, by applying an ice-cap several times a day for six or eight minutes.

Dr. Muller has cured a palsy of the Nervi optici, from the measles, by applying the Naphta phosphorata.

Dr. Frank communicates divers observations made in Egypt upon the efficacy of the Acidum Sacchari crudi (Vinaigre de Sucre) against the scurvy. The French general-in-chief Menou ordered the raw sugar in store, for the distillation of spirits for the use of the army; but the fermentation succeeded very imperfectly, and only a very small quantity was obtained: however the residue began to ferment a second time, and turned sour. This Acidum Sacchari contained but a very small part of alcohol, and also a little acid, but a still greater quantity of saccharine parts; and, from its resemblance in taste to the mixture of wine, sugar, and succus aurantiorum, recommended by Dr. Lind in scorbutic cases, Dr. Frank prescribed it to the very numerous patients labouring under that disorder. Some, being in the very last stage of it, took eight ounces every day, which dose was afterwards increased. Divers patients, nearly on the point of death, were saved by the use of it; and, of 400 patients, only 18 died.

Dr. Purlitz relates the following case of insanity, that took place five days after a palsy of the extremities, which had grown worse by applying evacuating remedies. He prescribed Tinctura Strammonii, prepared from four ounces of Spanish wine, half an ounce of alcohol, and one ounce of sem. strammonii; began with six drops twice a-day, which he afterwards raised to twenty-five drops. The remedy produced a sensible amendment, but not fully; and he was obliged to proceed to the Belladonna and Gratiola dissolved in Aqua Lauro-cerasi, according to Hufeland's method. Twenty-five drops were given three times a day of this mixture; and in about a fortnight the patient could be left without a guard. It is remarkable that the Tinct. Stramm. in this case produced salivation.

According to Dr. Hohnbaum's observations, the poisonous effects of the arsenic is to be distinguished from the inflammation of the stomach by two phenomena, that never occur in the latter, viz. an involuntary evacuation of urine and stool. In cases of a genuine genuine inflammation of the stomach, an obstruction of the bowels takes place; and an involuntary secretion of urine has never yet been observed in that complaint, on the contrary ischuria seems rather to take place. Both these symptoms, of course, sufficiently distinguish the forms of these two complaints.

Amongst the numerous forms of disease which, in the year 1815, appeared in the Vienna Hospital for sick children, receiving several thousands of patients, Dr. Goelis, the Director, observed one most nearly approaching to the transudative inflammation of the membrana cavi cerebri, and their venes, yet still distinguishing itself so much from it, particularly by its rapid progress, that he thought it deserving of a particular inquiry. The most careful dissection of the first case convinced this diligent observer that this appearance originated in an inflammation of the membrane of the medulla spinalis, which complaint had hitherto been considered by pathologists as barely possible, but whose actual existence had been scarcely known by physicians. Dr. Goelis has distinguished it by the appropriate name of Spinodorsitis.

Having observed the complaint seven times with unabated attention, he has thereby been enabled to delineate a faithful image of it; which, on account of the rarity of this important subject, and the perspicuity of the smallest indications, deserves our best thanks.

This complaint always appeared in the beginning with a considerable synocha, in most cases attended by general spasms in the extremities. From the first beginning of the fever, the posture of the patient was that of lying on his back, and a kind of tetanus contracted the muscles of the neck, back, and thighs; whereby the head was drawn backwards, the spine hollowed, and the legs pressed together, stiff and strained. The upper arms, by a similar spasm, were pressed close to the trunk, whilst the lower part moved automatically to the chin, mouth, or abdomen; and instantaneous convulsions, as if caused by electricity, shook the whole body. These motions gradually brought the patient down to the foot of the bed; meanwhile the marks of the most acute pain were imprinted on the high-red and burning-hot face, a pain which, on the least motion, particularly sideways, or on bending the feet upwards, or separating them, was expressed by pitiful cries. The patients of infant age enjoy but little sleep, delirium and sudden starts interrupting it. Children of larger growth, when awake, complain, with bitter cries, of a pain in their back, of an incapacity of moving their hands and feet, and the impossibility of turning themselves about.

The eye wanders about in every direction, the pupil much contracted, and more sensible; the nose dry, and the nostrils move on breathing, but the point always remains white, be the red colour of the face ever so high. The cavity of the mouth is red, the tongue moist, commonly covered with white; but even in the few cases of a clean tongue, the appetite ceases, though without a direct aversion to food. The thirst is tormenting, and insatiable; and, though the patient vomit up whatever they have swallowed so greedily under the most violent pains, yet they eagerly grasp at more drink. They almost dissolve in sweat, whilst, on the other hand, urine is voided scarce once or twice in twenty-four hours, and in small quantity. Pre-eminently distinguished before all analogical sufferings of the brain, and its component parts, is the increased irritability of the Tractus intestinorum, as frequently the smallest doses of calomel produce a diarrhœa, which sometimes even takes place without it. Notwithstanding the increased irritability of the pupillæ, the eye discerns every object presented, and the patients grasp at it; but if the inflammation of the medulla spinalis mounts, and attacks the substance of the cerebellum, and the brain itself, a true palsy of the pupilla, and even of the retina, takes place. If, with these symptoms of inflammation, a cough is accidentally combined, the sufferings of the patient are terrible, as the spasmodic stiffness of the neck becomes an excruciating obstacle to the muscles co-operating in coughing.

These are, according to Dr. Goelis's observations, the most essential symptoms defining this dreadful disorder, dreadful, as, in the greatest number of cases, it proves mortal, under convulsions, and, for the most part, apoplexy, after eighteen, twenty-four, or thirty-six hours at farthest. Sometimes it assumes the form of hydrops acutus cavi medullæ spinalis, and brings on inevitable death in a fortnight. However Dr. Goelis's success in snatching four patients of this Spinodorsitis from imminent danger, and restoring them again to life, is very encouraging. The indication pointed to all the means of subduing local inflammation. For this purpose, after a previous venesection in general, where the constitution of the patient made it requisite, he caused leeches to be applied in the nape of the neck, and on both sides of the back bone in the regions of the back and loins; or applied cupping-glasses, and a vesicatory an inch in breadth, from the third and fourth vertebræ colli to the os sacrum. He prescribed diluting, emollient, and composing beverage, of the infusion of marsh mallow, salep roots, &c. luke-warm, and in considerable quantity, to restore the secretion of the urinary organs, and the excretions, to their proper form, with now and then a few table-spoonfuls of a mixtura oleosa cum syrupo diacodii. Regard was had to the evacuations by stools; and where these did not follow, the calx mercurii, and, amongst these, calomel, in the smallest doses, rendered the most essential services. With regard to diet and regimen, Dr. Goelis directed the temperature to be kept rather cool than warm: children of a larger growth were only now and then to take a little mucilaginous broth; sucking infants the breast of the mother or nurse, whose food was carefully adapted to the condition of the patient.

By pursuing this method, Dr. Goelis has cured four patients of this Spinodorsitis; but in all of them the symptoms of fever continued till the eight or nine days. With every mark of genuine inflammation, it had killed others before the end of the second day. In one case, where, by a real transudation of the lymph and serum, a hydrops acutis spinæ dorsi had taken place in the cavity of the columna vertebralis, the disease terminated fatally after the fourteenth day.

If Dr. Goelis fulfils his promise, this Spinodorsitis, the Hydrops acutus medullæ spinalis, the Hydrocephalus chronicus, and Hydrops chronicus columnæ vertebralis, will constitute the contents of the second volume of his Practical Treatises upon the principal Disorders of Childhood, to which he intends adding, Observations upon the Chronic Induration of the Cutis tensa. This last disease ought to give a higher value to his work, as, notwithstanding that its existence is no longer doubtful, it has been almost entirely passed over by authors, and is hardly known by name to many physicians. Dr. Goelis was the first that discovered its nature to be of syphilitic origin, and, with a bold hand, combated it as such, by which he had the great satisfaction of so much lessening its former mortality, that, according to his diary, and the testimony of eye-witnesses, scarce five out of forty fell a sacrifice to it. Whoever, therefore, takes delight in our art, and patronises infancy, for which the medical art has hitherto not sufficiently exerted itself, will eagerly look forward to a work containing the nearer traits of this singular disorder, and announcing means for its fundamental cure, which shows the ways by which Dr. Goelis succeeded in finding out its most powerful antidote, mercury. Previous to the appearance of this second volume, a small Collection of remarkable Cases of Diseases, their origin and cure, will go through the press.

The Norway Society of Sciences has proposed, for 1815, the following prize Question, for her large silver medal:—Copiosa & dilucida historia pestiferi istius morbi vulgo *atra mors* dicti, eorum que malorum, quæ exinde ad Norwegiam redundarint.

The prize Question proposed by the Copenhagen University, for 1814, was—Catarohi explicatur theoria.

---

Mr. Laurence, junior surgeon at St. Bartholomew's, is delivering, with great satisfaction to his hearers, a public Course of Lectures on Comparative Anatomy, at the College of Surgeons. His first, or introductory lecture, was an elegant historical epitome. In his second he entered very much at large on the unfounded theories concerning the vital principle, and showed the propriety of Mr. Hunter's doctrine, that we can only trace the actions of life, without expecting to arrive at its cause.

---

Dr. Squire will, on Tuesday, April 2d, begin a Course of Lectures on the Principles and Practice of Midwifery, including the Diseases of Women and Children.

Dr.

Dr. Adams is preparing for the press, Memoirs of the Life, Doctrines, and Opinions of the late John Hunter, founder of the Hunterian Museum, at the College of Surgeons in London. These memoirs are carefully collected from authentic documents and anecdotes, and also from the writings, lectures, and conversations of the deceased.

---

Dr. Pinckard has just published a new edition of his "Notes on the West Indies" (including observations relative to the Creoles and Slaves of the Western Colonies, and the Indians of South America; with remarks upon the Seasoning or Yellow Fever of Hot Climates): with additional Letters from Martinique, Jamaica, and St. Domingo; also, further remarks on the Yellow Fever, and a Plan for the Emancipation of the Slaves in the West Indies.

---

Dr. C. H. Parry, of Bath, has just published an Inquiry into the Nature, Cause, and Varieties of the Arterial Pulse.

---

Dr. G. E. Male, of Birmingham, has just published, an Epitome of Juridical or Forensic Medicine.

---

### REPORT OF DISEASES.

The vernal constitution of the atmosphere evinces itself more strongly than ever in the prevailing diseases. Inflammation, in every form and in every organ, is as prevalent as ever; and hæmorrhage very frequent. The former requires the most prompt assistance, and, if relieved by ordinary bleeding, the consequence is only a suspension of the complaint, which returns with increased violence. Much is gained, however, by the first bleeding, for, though insufficient to remove the inflammatory disposition, it arrests the fatal symptoms, and allows the patient a respite till a practitioner of a more decided character is consulted.

Hæmorrhages from the nose or anus have been very profuse; but the patients have soon recovered. They have, in some instances, occurred in internal parts after symptoms of high inflammation in the thorax or lower viscera. The patient and the physician have fancied a favourable termination of the business, finding nothing to contend with but what they consider *typhoid symptoms*. On a sudden the patient, apparently convalescent, has breathed his last. On examining the body, profuse hæmorrhage has been discovered in one or other of the cavities.

Scarlet-fever has been very common among the new arrivals,—chiefly the servants who, for the first time, have visited the metropolis. The small-pox is more frequent than it ought to be. In London vaccination gains in reputation. We have heard less of the measles for the last month.

Meteorological

### METEOROLOGICAL REGISTER.

### *From February the 25th, to March the 26th,* 1816.

Kept by C. BLUNT, Philosophical Instrument Maker, No. 38, Tavistock-Street, Covent-Garden.

| Moon. | Day. | Wind. | Barometrical Pressure. Max. | Min. | Mean. | Temperature. Max. | Min. | Mean. | |
|---|---|---|---|---|---|---|---|---|---|
| | 26 | W | 29 98 | 29·94 | 29 96 | 49 | 27 | 38· | Fair |
| | 27 | W | 29 97 | 29 91 | 29·94 | 48 | 25 | 36·5 | Fair |
| ● | 28 | NW | 29 98 | 29 97 | 29·97 | 47 | 26 | 36·5 | Fair |
| | 29 | N | 29·98 | 29·97 | 29 97 | 47 | 26 | 36 5 | Fair |
| | 1 | N | 29·96 | 29 94 | 29 95 | 49 | 28 | 38·5 | Fair |
| | 2 | SW | 29 93 | 29 90 | 29 91 | 50 | 29 | 39·5 | Fair |
| | 3 | SE | 29·90 | 29·88 | 29 89 | 47 | 33 | 40· | Fair |
| | 4 | NW | 29 86 | 29·80 | 29·83 | 48 | 27 | 37·5 | Snow |
| | 5 | NW | 29·71 | 29·48 | 29·59 | 48 | 33 | 40 5 | Snow |
| | 6 | W | 29 46 | 29·40 | 29·43 | 49 | 40 | 44·5 | Fair |
| ◐ | 7 | SW | 29 40 | 29·36 | 29·38 | 48 | 36 | 42· | Rain |
| | 8 | S | 29·25 | 29·20 | 29·22 | 46 | 36 | 41· | Rain |
| | 9 | SE | 29·26 | 29·14 | 29·20 | 47 | 38 | 42 5 | Rain |
| | 10 | SW | 29·39 | 29 29 | 29·34 | 49 | 36 | 42·5 | Fair |
| | 11 | SW | 29·70 | 29·40 | 29 55 | 51 | 38 | 44·5 | Fair |
| | 12 | W | 30·26 | 30·10 | 30·18 | 52 | 37 | 44·5 | Rain |
| ○ | 13 | SW | 30·30 | 30·25 | 30·27 | 51 | 36 | 43 5 | Fair |
| | 14 | W | 30 60 | 30·40 | 30·50 | 52 | 34 | 43· | Rain |
| | 15 | W | 29·80 | 29·70 | 29·75 | 52 | 34 | 43· | Rain |
| | 16 | NW | 29·58 | 29·56 | 29·57 | 53 | 35 | 44· | Rain |
| | 17 | NW | 29·62 | 29 60 | 29·60 | 54 | 36 | 45· | Rain |
| | 18 | NW | 29 80 | 29·70 | 29·75 | 53 | 37 | 45· | Fair |
| | 19 | NW | 29·95 | 29·90 | 29·93 | 51 | 38 | 44 5 | Fair |
| ◑ | 20 | W | 30· | 30· | 30· | 51 | 38 | 44·5 | Rain |
| | 21 | W | 30·20 | 30· | 30·10 | 50 | 37 | 43·5 | Rain |
| | 22 | SW | 30·26 | 30·24 | 30·25 | 50 | 36 | 43· | Fair |
| | 23 | SE | 30 28 | 30·25 | 30·27 | 49 | 35 | 42· | Fair |
| | 24 | SE | 30·24 | 30·19 | 30·21 | 50 | 34 | 42· | Cloudy |
| | 25 | E | 30·20 | 30·17 | 30·18 | 49 | 35 | 42· | Fair |
| | 26 | SE | 30·21 | 30·18 | 30·20 | 48 | 35 | 41·5 | Fair |

RESULTS.

Mean barometrical pressure of the month 29·86
Maximum 30·60, wind at W
Minimum 29·14, ——— SE

Mean temperature of the month 41·7 deg.
Maximum 54, wind at NW
Minimum 25, ——— W

*Scale exhibiting the prevailing Winds during the Month.*

| N | NE | E | SE | S | SW | W | NW |
|---|---|---|---|---|---|---|---|
| 2 | 0 | 1 | 5 | 1 | 6 | 8 | 7 |

| | Mean barometrical pressure. | Mean temperature. |
|---|---|---|
| From the last quarter on the 20th Feb. to the new moon on the 28th | 30 08 | 40·12 |
| ——— new moon on the 28th Feb. to the first quarter on the 7th March | 29·74 | 39 87 |
| ——— first quarter on the 7th, to the full moon on the 13th | 29·62 | 43·08 |
| ——— full moon on the 13th, to the last quarter on the 20th | 29·87 | 44·14 |

